# Over-expression of 14-3-3zeta is an early event in oral cancer

**DOI:** 10.1186/1471-2407-7-169

**Published:** 2007-09-02

**Authors:** Ajay Matta, Sudhir Bahadur, Ritu Duggal, Siddhartha D Gupta, Ranju Ralhan

**Affiliations:** 1 Department of Biochemistry, All India Institute of Medical Sciences, Ansari Nagar, New Delhi -110029, India; 2 Department of Otorhinolaryngology, All India Institute of Medical Sciences, Ansari Nagar, New Delhi -110029, India; 3 Department of Dental Surgery, All India Institute of Medical Sciences, Ansari Nagar, New Delhi -110029, India; 4 Department of Pathology, All India Institute of Medical Sciences, Ansari Nagar, New Delhi -110029, India

## Abstract

**Background:**

The functional and clinical significance of 14-3-3 proteins in human cancers remain largely undetermined. Earlier, we have reported differential expression of 14-3-3ζ mRNA in oral squamous cell carcinoma (OSCC) by differential display.

**Methods:**

The clinical relevance of 14-3-3ζ protein in oral tumorigenesis was determined by immunohistochemistry in paraffin embedded sections of oral pre-malignant lesions (OPLs), OSCCs and histologically normal oral tissues and corroborated by Western Blotting. Co-immunoprecipitation assays were carried out to determine its association with NFκB, β-catenin and Bcl-2.

**Results:**

Intense immunostaining of 14-3-3ζ protein was observed in 61/89 (69%) OPLs and 95/120 (79%) OSCCs. Immunohistochemistry showed significant increase in expression of 14-3-3ζ protein from normal mucosa to OPLs to OSCCs (p_trend _< 0.001). Significant increase in expression of 14-3-3ζ protein was observed as early as in hyperplasia (p = 0.009), with further elevation in moderate and severe dysplasia, that was sustained in OSCCs. These findings were validated by Western blotting. Using Co-immunoprecipitation, we demonstrated that 14-3-3ζ protein binds to NFκB, β-catenin and Bcl-2, suggesting its involvement in cellular signaling, leading to proliferation of oral cancer cells.

**Conclusion:**

Our findings suggest that over-expression of 14-3-3ζ is an early event in oral tumorigenesis and may have an important role in its development and progression. Thus, 14-3-3ζ may serve as an important molecular target for designing novel therapy for oral cancer.

## Background

Squamous cell carcinoma of Head and Neck, including oral cavity, is a major problem worldwide. Oral squamous cell carcinoma (OSCC) is often preceded by development of oral pre-malignant lesions (OPLs), of which on an average about one percent transform into cancer annually [1]. Molecular markers that can identify OPLs at high risk of malignant transformation and predict the course of disease remain to be unequivocally proven. Currently, the most important conventional prognostic factors for survival of OSCC patients are histological tumor grade and tumor stage at the time of diagnosis, including depth of tumor invasion and involvement of regional lymph nodes. In addition to these clinicopathological parameters, molecular markers are being intensively sought and validated for oral cancer. These new markers are being examined for their diagnostic and prognostic impact, and even therapeutic implications as novel drug targets. In search of such novel targets, we recently reported increased expression of 14-3-3ζ in oral cancer using Differential Display [2].

14-3-3 proteins are highly conserved eukaryotic proteins that are involved in most of the cellular processes, including regulation of several metabolic pathways, redox regulation, transcription RNA processing, protein synthesis, folding and degradation, cell cycle, apoptosis, cytoskeletal organization and cellular trafficking by binding to phosphorylated sites in diverse target proteins (over 300 phosphoproteins identified) [3-7]. In mammalian cells, seven different isoforms have been identified (ζ,β,γ,ε,σ,η and θ) with each isoform having distinct tissue localization and function. 14-3-3 proteins can form homodimers or heterodimers that allow them to function as an adapter, linker, scaffold or coordinator in assembling signaling complexes [8-10]. 14-3-3 proteins associate with a number of different signaling proteins including MEKK1, and PI-3 kinase [11,12], apoptosis regulatory proteins ASK-1 and tumor suppressor p53 [13-15], transcription regulatory protein FKHRL1 and DAF-16 and histone deacetylase [16-18]. 14-3-3 proteins promote cell survival through their interactions with signaling proteins such as EGFR, Raf-1, the pro-apoptotic protein BAD (Bcl-2/Bcl-X_L_-antagonist causing cell death) and the cell cycle phosphatase cdc25 [19,20]. Furthermore, 14-3-3s may have multiple roles in connecting signaling pathways to the regulation of actin-based cellular changes in cytoskeleton and cell motility [21]. Recent studies have suggested that 14-3-3 proteins are potential oncogenes [22].

Herein we hypothesized an association of 14-3-3ζ with oral tumorigenesis. To test this hypothesis immunohistochemical analysis of 14-3-3ζ protein was carried out in paraffin embedded sections of human oral normal tissues, OPLs and OSCCs and its correlation was determined with development and progression of oral cancer. Co-immunoprecipitation assays were carried out to determine its involvement in different signaling pathways leading to increased cell proliferation.

## Methods

### Patients

Institutional Human Ethics Committee approved this study prior to its commencement. Biopsies of oral pre-malignant lesions (leukoplakia, n = 89, including hyperplasias (n = 49) and dysplasias (n = 41) were collected from patients attending Outpatient Department of Department of Otorhinolaryngology, All India Institute of Medical Sciences, New Delhi, India. Surgically resected specimens of human OSCCs (n = 120) and non-malignant tissues (taken from a distant site) were collected from patients undergoing curative oral cancer surgery at Department of Otorhinolaryngology, after obtaining prior written consent of the patients. Normal oral tissues with no evidence of OPLs or OSCCs, were also collected from patients attending the Dental Outpatient Department for tooth extraction. After excision, tissues were immediately snap frozen in liquid N_2 _and stored at -80°C till further use and one piece was collected in 10% formalin and embedded in paraffin for histopathological analysis. The clinical and pathological data were recorded in a pre-designed proforma. These included clinical TNM staging (tumor, node, metastasis based on Union International Center le Cancer TNM classification of malignant tumors 1998), site of the lesion, histopathological differentiation, age, gender and consumption of betel quid, areca nut and tobacco.

### Cell culture and immunofluorescence

Human oral squamous carcinoma cell line, HSC2, was used in this study. Cells were grown in monolayer cultures in Dulbecco's modified eagle medium (DMEM-F12) supplemented with 10% Fetal bovine serum (FBS) (Sigma-Aldrich, St. Louis, MO), 100 μg/ml Streptomycin and 100 U/ml Penicillin in a humidified incubator (5% carbon-dioxide, 95% air) at 37°C. To study the sub-cellular localization of 14-3-3ζ protein, immunofluorescence (IF) was determined in oral cancer cells. For IF, 5 × 10^4 ^cells were plated on cover slips and grown for 48 hrs. Thereafter, cells were washed with Phosphate buffered saline, (PBS, 0.01 M, pH = 7.2) and fixed in acetone: methanol mixture(1:1) for 20 minutes. Cells were washed and permeablized with 0.2% Tween in PBS followed by blocking with 2% BSA for 1 hr. These cells were then incubated with rabbit polyclonal anti-14-3-3ζ antibody (Santa Cruz Biotechnology, CA) at 4°C O/N. Expression of 14-3-3ζ protein was determined by streptavidin-conjugated-FITC labeled secondary antibody (DAKO Cytomation, Denmark) as described earlier [23].

### Immunohistochemical staining

Paraffin embedded sections (5 μm) of human oral normal tissues (n = 66), OPLs (Leukoplakia, n = 89) and OSCCs (n = 120) were collected on gelatin coated slides. For histopathological analysis, representative sections were stained with hematoxylin and eosin and serial sections were used for immunostaining as described by us previously [2]. Briefly, the sections were deparaffinized in xylene, hydrated and pretreated in a microwave oven in citrate buffer [0.01 M (pH 6.0)] for antigen retrieval. The sections were incubated with hydrogen peroxide (0.3% v/v) in methanol for 20 minutes to quench the endogenous peroxidase activity. Non-specific binding was blocked with 1% bovine serum albumin (BSA) in Phosphate buffered saline (PBS, 0.01 M, pH = 7.2) for 1 h. Thereafter, slides were incubated with rabbit polyclonal anti-14-3-3ζ antibody (Santa Cruz Biotechnology Inc.) at a dilution of 1:200, for 16 hrs at 4°C and washed with PBS. The primary antibody was detected using strep-avidin-biotin complex using DAKO LSAB plus kit (DAKO Cytomation, Denmark) and diaminobenzidine as chromogen. All incubations were performed at room temperature in a moist chamber. Slides were washed with Tris-buffered saline (TBS, 0.1 M, pH = 7.4), 3 times after every step. Finally, the sections were counterstained with Mayer's hematoxylin and mounted with D.P.X mountant. In negative controls, the primary antibody was replaced by non-immune mouse IgG of the same isotype to ensure specificity. OSCC tissue sections with known immunopositivity for 14-3-3ζ protein as reported earlier [2], were used as positive control in each batch of sections analyzed.

### Evaluation of immunohistochemical staining

14-3-3ζ immunoreactivity was evaluated in five areas of the tissue sections as described by us previously [2] and specific staining in epithelial cells was defined as positive staining. Tissue sections were graded as 14-3-3ζ immunopositive if >10% of epithelial cells showed moderate/intense staining in cytoplasm and/or plasma membrane and/or nucleus by two of us independently who were blinded to clinical outcome i.e. the slides were coded and pathologist did not have prior knowledge of the local tumor burden, lymphonodular spread and grading of the tissue samples while scoring the immunoreactivity.

### Statistical analysis

The immunohistochemical data was subjected to statistical analysis using SPSS 10.0 software. The relationship between 14-3-3ζ protein expression and clinicopathological parameters was tested by Chi-Square and Fischer's exact test. Two sided p values were calculated and p ≤ 0.05 was considered to be significant.

### Immunoblot analysis of 14-3-3ζ in oral normal tissues, OPLs and OSCCs

Whole cell lysates were prepared from 5 normal oral tissues with no evidence of malignancy, 5 OPLs (3 hyperplasias and 2 dysplasias) and 8 OSCCs. Frozen tissue samples were homogenized and lysed in IP lysis buffer containing 50 mM Tris-Cl (pH 7.5), 150 mM NaCl, 10 mM MgCl_2_, 1 mM ethylene diamine-tetracetate (pH 8.0), 1% Nonidet P-40, 100 mM sodium fluoride, 1 mM phenylmethylene-sulphonylfluoride and 2 μl/ml protease inhibitor cocktail (Sigma). Protein concentration was determined using Bradford reagent (Sigma) and equal amount of protein (80 μg/lane) from normal oral tissues, OPLs and OSCCs was resolved on 12% sodium dodecyl sulphate(SDS)-polyacrylamide gel. The proteins were then electro-transferred onto Polyvinylidenedifluoride (PVDF) membrane. After blocking with 5% non-fat powdered milk in Tris-buffered saline (TBS, 0.1 M, pH = 7.4), blots were incubated with anti-14-3-3ζ antibody (1:500 dilution) at 4°C overnight. Protein abundance of α-tubulin served as a control for protein loading, and was determined with mouse monoclonal anti-α-tubulin antibody (Santa Cruz Biotechnology). Membranes were incubated with secondary antibody, HRP-conjugated rabbit/mouse anti-IgG (DAKO Cytomation, Denmark), diluted at an appropriate dilution in 1% BSA, for 2 hrs at room temperature. After each step blots were washed thrice with Tween (0.2%)-Tris-buffer saline (TTBS). Protein bands were detected by Enhanced chemiluminescence method (ECL, Santa Cruz Biotechnology, CA) on XO-MAT film.

### Co-immunoprecipitation

Oral cancer cells, HSC2 were rinsed in ice-cold PBS and lysed in IP lysis buffer. Lysates were incubated on ice for 30 minutes and cell debris was removed by centrifugation. Thereafter, lysates were pre-cleared by adding 20 μl of protein A-Sepharose(GE Healthcare Biosciences, Sweden) followed by incubation with polyclonal 14-3-3ζ and NFκB antibodies or monoclonal β-catenin and Bcl-2 antibodies (at a dilution of 1:100, Santa Cruz Biotechnology) over-night on a rocker at 4°C. Immune complexes were pulled down by incubating with Protein A-Sepharose for 4 hrs at 4°C followed by washing with ice-cold lysis buffer 4-5 times, to eliminate non-specific interactions. Protein A-Sepharose bound immune-complexes were then resuspended in Laemelli sample buffer, pH = 7.4 (10 mMTris, 10% v/v glycerol, 2% w/v SDS, 5 mM EDTA, 0.02% bromophenol blue and 6% β-mercaptoethanol), boiled for 5 minutes and analyzed by western blotting using specific antibodies as described above. In negative control, primary antibody was not added to the cell lysate.

### Motif search

To check the presence of 14-3-3 binding motif, peptide sequences of 14-3-3ζ, NFκB, β-catenin and Bcl-2 were screened using ScanSite software available online.

## Results

### Immunohistochemical analysis of 14-3-3ζ in oral normal tissues, OPLs and OSCCs

**Figure 1:**
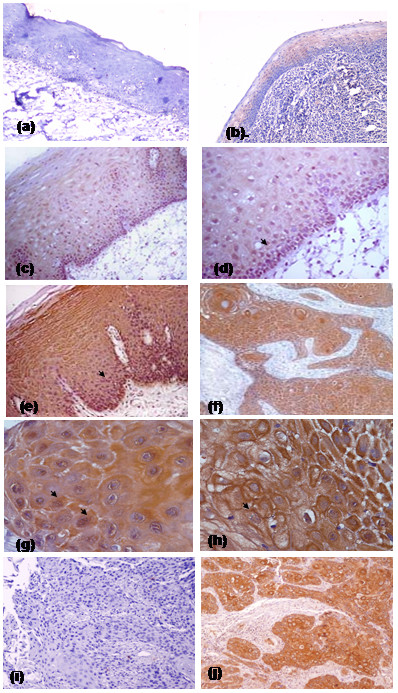
Immunohistochemical analysis of 14-3-3ζ in oral tissues. Paraffin embedded oral tissue sections were stained using anti-14-3-3ζ antibody as described in Materials and Method section. (a) Normal oral mucosa showing no detectable immunostaining of 14-3-3ζ protein (original magnification ×100). (b) Inflamed normal oral mucosa showing cytoplasmic immunostaining of 14-3-3ζ protein (original magnification × 100). (c) Oral hyperplasia showing cytoplasmic and nuclear immunostaining of 14-3-3ζ(original magnification × 100). (d) Oral hyperplasia showing nuclear and cytoplasmic immunostaining at higher magnification (original magnification × 400). (e) Dysplastic lesion showing cytoplasmic immunostaining in epithelial cells (original magnification × 200). (f) OSCC showing cytoplasmic and nuclear staining in tumor cells (original magnification × 100). (g) OSCC showing cytoplasmic and nuclear staining in tumor cells (original magnification × 400). (h) OSCC showing cytoplasmic and membranous staining in tumor cells (original magnification × 400). (i) Negative control showing no immunostaining of 14-3-3ζ protein in epithelial cells (original magnification × 100). (j) Positive control is a known esophageal squamous cell carcinoma showing cytoplasmic immunostaining of 14-3-3ζ in epithelial cells (original magnification × 100). Arrow shows nuclear staining in Panels 1d, 1e and 1 h and membranous staining in Panel 1 g respectively.

Significant increase in 14-3-3ζ expression was observed in different stages of oral tumorigenesis [viz. normal to hyperplasia, dysplasia and cancer (p_trend _< 0.001)] as revealed by Chi-Square trend analysis shown in Table 1. The results of immunohistochemical analysis of 14-3-3ζ protein in normal oral tissues, OPLs and OSCCs are summarized in Tables 2 and 3 respectively. No detectable or faint immunostaining of 14-3-3ζ was observed in epithelial cells in histologically normal oral tissues (Figure 1a), whereas 38% of the oral normal tissues that had inflammation showed cytoplasmic staining only (Figure 1b). Increased expression (cytoplasmic/membranous/nuclear) of 14-3-3ζ protein was observed in 33 of 48 (69%) hyperplasias [p = 0.009, OR = 2.8], 52% of these lesions showed cytoplasmic expression only (Figure 1c). In addition, rapidly proliferating epithelial cells in the basal and parabasal layers of hyperplastic lesions, showed distinct nuclear immunopositivity (Figure 1d). Of the 41 dysplasias analyzed in this study, 67% showed immunopositivity for 14-3-3ζ protein as shown in Figure 1e. Among the dysplasias analyzed, an increase in overall 14-3-3ζ positivity was observed from mild to moderate dysplasia that was sustained in severe dysplasia. Furthermore, there was a difference in the sub-cellular localization of 14-3-3ζ in dysplasias in comparison with the normal oral tissues. In the normal oral tissues, the protein was localized in the cytoplasm, whereas in the mild dysplasia membrane immunopositivity was also observed, that increased in moderate dysplasia. In addition, nuclear localization was also observed in moderate dysplasia, which increased in severe dysplasia. However, the number of cases showing membrane or nuclear positivity in each sub-category was small for statistical analysis. Nevertheless, these observations may be important in view of the role of 14-3-3ζ as an adapter molecule in various cellular processes. The biological impact of differences in the sub-cellular localization of 14-3-3ζ in normal, OPLs and OSCCs on the development and progression of oral cancer remains to be determined in a larger study.

**Table 1:** Expression of 14-3-3ζ protein in Oral Tissues

**Tissue Type**	**Total no. of cases**	**14-3-3ζ Overall Positivity**		**p-value**	**OR (95% CI)**
	**(N)**	**N**	**(%)**		
**NORMAL**	66	25	(38)		
**OPLs**	89	61	(69)	<0.001*^a^	**3.5(1.8–6.7)**
**OSCCs**	120	95	(79)	< 0.001*^b^	**6.2(3.2–12.1)**

*a Normal vs. OPLs; *b Normal vs. OSCCs.

# Normal/Hyperplasia/Dysplasia/SCC Chi Square trend Analysis, p < 0.001.

**Table 2:** Analysis of 14-3-3ζ protein expression in Oral Pre-Malignant lesions: Correlation with clinicopathological parameters

**Clinicopathological Features**	**Total Cases**	**14-3-3 zeta overall Positivity**		**14-3-3 zeta Cytoplasmic Positivity**		**14-3-3 zeta Cytoplasmic & Membrane Positivity**		**14-3-3 zeta Cytoplasmic & Nuclear Positivity**	
	N	n	(%)	n	(%)	n	(%)	n	(%)
**Normal**	66	25	(38)	25	(38)	0		0	
**OPLs**	89	61	(69)	46	(52)	8	(9)	7	(8)
**Differentiation**									
Hyperplasia^a^	48	33	(68)	25	(52)	4	(8)	4	(8)
Dysplasia	41	28	(67)	21	(51)	4	(10)	3	(7)
Mild Dysplasia^b^	19	10	(53)	9	(47)	1	(5)	0	
Moderate Dysplasia	14	11	(78)	8	(57)	2	(14)	1	(7)
Severe Dysplasia	8	7	(87)	4	(50)	1	(12)	2	(25)
**Habits**									
**Non consumer**	12	7	(58)	5	(42)	1	(8)	1	(8)
**Tobacco consumer**	77	54	(70)	41	(53)	7	(9)	6	(8)

**# OPLs: **Age range:18–74 yrs, Median age, 44 yrs ; No. of Males :79 ; Females:10; Non consumers :12; Tobacco consumers (including betel quid usage): 77 (p > 0.05);

^a^**Normal/Hyperplasia, p = 0.009;**

^b^Mild/Moderate/Severe Dysplasia, p = 0.123

**Table 3:** Analysis of 14-3-3 zeta protein expression in OSCCs: Correlation with clinicopathological parameters

**Clinicopathological Features**	**Total Cases**	**14-3-3 zeta overall Positivity**		**14-3-3 zeta Cytoplasmic Positivity**		**14-3-3 zeta Cytoplasmic & Membrane Positivity**		**14-3-3 zeta Cytoplasmic & Nuclear Positivity**	
	N	n	(%)	n	(%)	n	(%)	n	(%)
**OSCC**	120	95	(79)	77	(65)	8	(6)	10	(8)
**Differentiation**									
WDSCC	67	51	(76)	43	(64)	4	(6)	4	(6)
MDSCC	47	39	(83)	29	(61)	4	(8)	6	(12)
PDSCC	6	5	(83)	5	(83)	0		0	
**Tumor Stage**									
T_1_	20	17	(85)	15	(75)	1	(5)	1	(5)
T_2_	36	24	(66)	18	(50)	2	(5)	4	(11)
T_3_	32	27	(84)	19	(60)	5	(15)	3	(9)
T_4_	32	27	(84)	25	(78)	0		2	(6)
**Nodal Status**									
N^-^	66	51	(77)	43	(65)	4	(6)	4	(6)
N^+^	54	44	(81)	34	(63)	4	(7)	6	(11)
**Habits**									
Non consumer	16	12	(75)	10	(62)	1	(6)	1	(6)
Tobacco consumer	104	83	(80)	67	(64)	7	(7)	9	(9)

**# OSCCs: **Age Range: 29–75 yrs, Median age, 53 yrs; No. of Males: 95; Females:25 ; Non consumers :16; Tobacco consumers (including betel quid usage):104 (p > 0.05) No correlation was observed between 14-3-3ζ protein expression and histopathological grading, tumor stage and nodal status of OSCCs.

Among OSCCs, overall immunopositivity of 14-3-3ζ protein (cytoplasmic and/or membrane/nuclear) was observed in 95 of 120 (79%) cases analyzed as shown in Figure 1f. Nuclear staining of 14-3-3ζ protein was observed in 10 of 120 OSCCs (Figure 1g), whereas 6% of OSCCs showed membranous expression in addition to cytoplasmic staining (Figure 1h). No immunostaining was observed in tissue sections used as negative controls where primary antibody was replaced by isotype specific IgG (Figure 1i), while positive control showed cytoplasmic expression of 14-3-3ζ protein (Figure 1j). Over-expression of 14-3-3ζ protein in OPLs/OSCCs did not show any correlation with age, gender, betel quid and tobacco consumption habits, differentiation, tumor stage or nodal status of OSCC patients (Table 2).

### Sub-cellular localization of 14-3-3ζ by immunofluorescence in oral cancer cells

**Figure 2:**
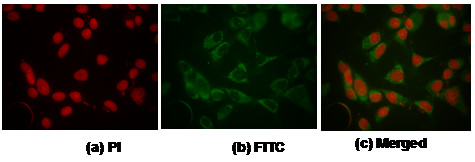
Immunofluorescence of 14-3-3ζ protein in oral cancer cells, HSC2. Cells were fixed and immunolabeled with anti-14-3-3ζ antibody followed by streptavidin-conjugated-FITC labeled secondary antibody (Green fluorescence) and nuclei were counterstained with propidium iodide (red fluorescence). (a) Nuclei showing red fluorescence of Propidium Iodide. (b) Green fluorescence in cytoplasm and nucleus of HSC2 cells. (c) Merged figure showing localization of 14-3-3ζ protein in oral cancer cells (a-c, original magnification × 200).

Immunofluorescence analysis was carried to validate the sub-cellular localization of 14-3-3ζ protein in oral cancer cells. Immunofluorescence analysis showed intense cytoplasmic and moderate nuclear expression of 14-3-3ζ in oral cancer cells, HSC2, as shown in Figure 2.

### Immunoblotting and co-immunoprecipitation

**Figure 3:**
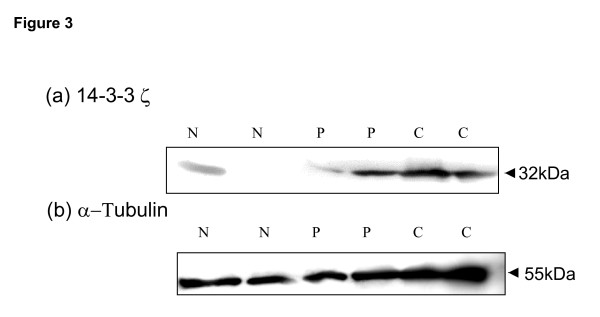
Immunoblot analysis of 14-3-3ζ in oral tissues. Equal amounts of whole cell lysates of normal oral tissues (N), OPLs (P) and OSCCs (C) from the same patients as used for IHC were resolved on 12% SDS-PAGE. Proteins were then electro-transferred on PVDF membrane followed by blocking with 5% non-fat milk O/N. Blots were incubated with anti-14-3-3ζ polyclonal antibody and protein expression was determined using Enhanced chemiluminescence method. Immunoblots of: (a) 14-3-3ζ protein and (b) α-tubulin, in normal oral tissue (N), oral pre-malignant lesion (P) and OSCC (C).

Further, to validate the over-expression of 14-3-3ζ protein in oral lesions, immunoblotting was carried out in the same tissue samples as used for immunohistochemical analysis. Increased expression of 14-3-3ζ was observed in OPLs and OSCCs as compared to normal oral tissues (Figure 3a). These findings are in concordance with the immunohistochemical data. α-tubulin served as an internal control for equal loading of protein in each well of SDS-PAGE (Figure 3b).

**Figure 4:**
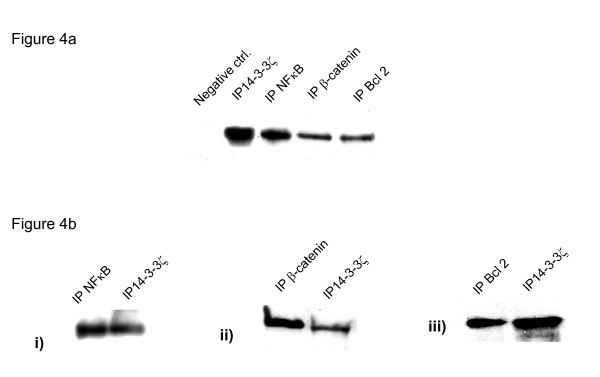
Immunoblot analysis after Co-immunoprecipitation assay. Immunoprecipitation assays of 14-3-3ζ, NFκB, β-catenin and Bcl-2 proteins were carried out in oral cancer cells, HSC2, as described in Materials and Method section. The immunoprecipitates were resolved on 12% SDS-PAGE and electro-transferred on PVDF membrane followed by blocking with 5% non-fat milk O/N. Blot was incubated with 14-3-3ζ antibody and protein expression was determined using Enhanced Chemiluminescence method. Figure 4a shows immunoblot analysis 14-3-3ζ, demonstrating the binding of 14-3-3ζ protein with NFκB, β-catenin and Bcl-2 proteins. However, no band of 14-3-3ζ was observed in the negative control. Similarly, reverse immunoprecipitation assays were carried out using specific antibodies for NFκB, β-catenin and Bcl-2 proteins. Figure 4b shows immunoblot analysis of: (i) NFκB; (ii) β-catenin and (iii) Bcl-2 protein confirming the binding of these proteins with 14-3-3ζ.

**Figure 5:**
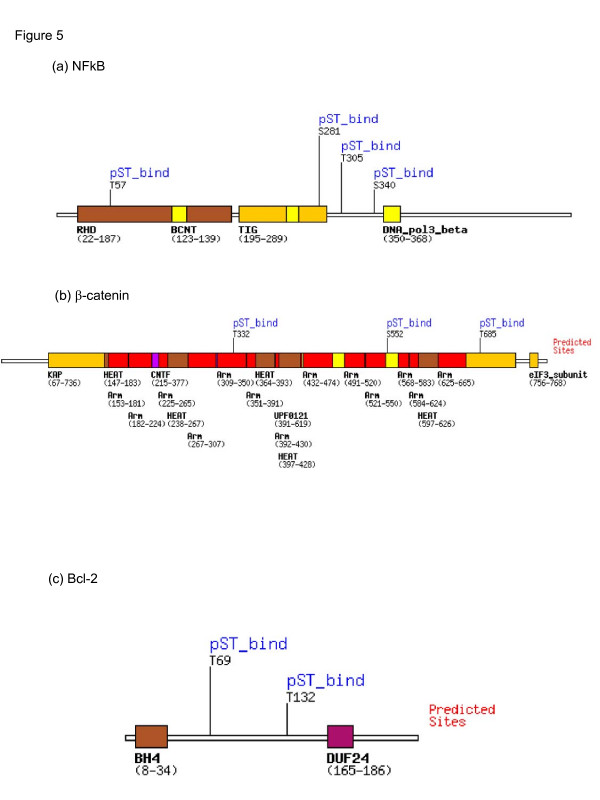
Motifscan of 14-3-3 binding motif Mode1. Using Scansite software , motif search showed presence of 14-3-3 binding motif, Mode 1 in (a) NFκB; (b) β-catenin and (c) Bcl-2 proteins.

As a first step in determining the functional significance of 14-3-3ζ in oral carcinogenesis, we identified its binding partners in oral cancer cells, HSC2, using co-immunoprecipitation (IP). Immunoprecipitation of 14-3-3ζ protein revealed its binding to p65 subunit of NFκB, β-catenin and Bcl-2 proteins (Figure 4a) suggestive of its involvement in inflammation, survival and proliferation of cancer cells. Reverse immunoprecipitation assays using specific antibodies for NFκB, β-catenin and Bcl-2 proteins confirmed their binding to 14-3-3ζ (Figure 4b). However, no band was observed in immunoblot analysis in negative controls. Motif search using Scansite software revealed the presence 14-3-3 binding motif, Mode 1 in NFκB, β-catenin and Bcl-2 proteins (Figure 5).

## Discussion

Herein, we demonstrate for the first time that over-expression of 14-3-3ζ occurs in pre-malignant stages of oral cancer, as early as in hyperplasia and elevated expression is sustained down the tumorigenic pathway. In hyperplastic and dysplastic tissues, epithelial cells in the rapidly proliferating basal layer showed nuclear staining in addition to cytoplasmic localization of 14-3-3ζ protein, suggesting that it may be involved in nuclear cytoplasmic shuttling of proteins in rapidly proliferating cells. Our immunofluorescence results also showed cytoplasmic and nuclear localization of 14-3-3ζ in oral cancer cells, HSC2, confirming the immunohistochemistry data. These findings are also supported by other studies which demonstrate abundant cytoplasmic and nuclear localization of several 14-3-3 isoforms, including 14-3-3ζ [24-26]. Hemert *et al*. [27] proposed that the nucleo-cytoplasmic shuttling of 14-3-3ζ in mammalian cells (Hela and HaCat cells) may have a role in regulation of cellular processes. Interestingly, the nuclear/cytoplasmic expression of 14-3-3ζ protein has also been proposed to be involved in cell cycle regulation and DNA replication during embryonic development in *Drosophila melanogaster, C. elegans *and *Xenopus laevis *[28-30]. Currently studies are underway to determine the functional significance of 14-3-3ζ localization in different sub-cellular compartments in oral cancer cells.

Major thrust in understanding the biology of oral cancer has been laid on unraveling the proteins involved in malignant transformation, while the early events involved in genesis of pre-neoplastic lesions remain largely obscure. However, in recent years there has been a paradigm shift in discovery of new drugs for cancer management. The focus has shifted from cancer treatment to prevention. It is becoming increasingly clear that attention needs to be focused on rational designing of chemopreventive agents directed towards specific molecular targets that are altered in early stages, prior to malignant transformation for effective intervention in the carcinogenic process. The upregulation of 14-3-3ζ at transcriptional and translational levels in early stages of oral tumorigenesis prompted us to hypothesize that 14-3-3ζ may be a link between chronic inflammation and cancer. To test this hypothesis, we carried out Co-immunoprecipitation assays to identify its binding partners in oral cancer cells. Our results demonstrated binding of 14-3-3ζ to NFκB, β-catenin and Bcl-2 in these cells. In this context, the interaction of 14-3-3ζ with NFκB is of major importance and suggestive of its probable role in linking inflammation and proliferation of oral epithelial cells. It is noteworthy that in our earlier study, we showed transcriptional upregulation and increased expression of NFκB and 14-3-3ζ in oral premalignant lesions (hyperplasia and dysplasia) and oral cancer [31]. Our present findings are also supported by the recent study of Aguilera *et al*. [32] which showed that 14-3-3 proteins facilitate the nuclear export of IκBα-p65 complex and are required for the appropriate regulation of NFκB signaling after treatment with tumor necrosis factor alpha (TNFα). Thus, nucleo-cytoplasmic shuttling of 14-3-3s may have a role to play in translocation of NFκB from cytoplasm to nucleus in presence of specific stimulus.

In an attempt to understand the functional significance of increased expression of 14-3-3ζ in oral carcinogenesis, we carried out co-immunoprecipitation assays to identify the binding partners of 14-3-3ζ and showed its binding to β-catenin. It is noteworthy that β-catenin, functioning as a major component of both Wnt signaling and cell-cell adhesion, plays a central role in cell proliferation, differentiation, polarity, morphogenesis, and development [33-35]. Interestingly, 14-3-3ζ, in association with β-catenin, has been shown to facilitate transactivation of β-catenin by AKT [36]. In turn, the transactivation of β-catenin increases the transcription of genes that promote tumor cell growth, such as *MYC *[37], *CCND1 *(which encodes *cyclin D*) [38,39] and *JUN *[40]. Using immunoprecipitation Fang *et al*. [41], demonstrated that phosphorylation of β-catenin at serine 552 leads to its dissociation from cell-cell contacts, increases its binding to 14-3-3ζ and its transcriptional activity. It is noteworthy that in a parallel study in our laboratory, we showed loss of membranous β-catenin and its nuclear accumulation to be adverse prognosticators in OSCC patients (Sawhney *et al*., personal communications). We have previously reported increased cyclin D1 protein expression in OPLs and OSCCs and proposed that deregulation of the p16/pRb/cyclin D1 pathway is an early event in the acquisition of dysplasia, but deregulation of both pRb and p53 pathways is associated with malignant transformation and adverse prognosis in oral tumorigenesis [42]. Taken together, the present study suggests that β-catenin is one of the targets of 14-3-3ζ that may be implicated in cellular pathways involved in increased cell proliferation.

Hyperproliferation of cancer cells is not only attributed to increase cell division but also to deregulation of apoptosis. In this context, we demonstrated the binding of 14-3-3ζ with Bcl-2 in oral cancer cells and propose that the interaction of 14-3-3ζ with Bcl-2 may result in its sequestration and consequent abrogation of apoptosis. Our findings are in accord with the well established role of 14-3-3 proteins in abrogation of apoptosis by sequestering Bcl-2 or its family members (Bad, Bax) [43-45]. In-depth analysis of these interactions is currently underway to elucidate their functional significance in oral carcinogenesis. Interestingly, over-expression of 14-3-3ζ has also been reported in esophageal squamous cell carcinomas [46], lung [47] and stomach cancer [48], supporting our findings in OSCCs and underscoring the importance of this protein in human cancers. In view of the above in depth analysis of the functional significance of 14-3-3ζ in oral carcinogenesis is warranted.

## Conclusion

This study demonstrates increased expression of 14-3-3ζ as early as in oral hyperplasia and its sustained expression down the carcinogenic pathway. Our results suggest involvement of 14-3-3ζ in cell signaling pathways involved in inflammation, cell proliferation and abrogation of apoptosis during oral carcinogenesis. Hence, the upregulation of 14-3-3ζ expression in early pre-neoplastic stages of oral tumorigenesis, both at transcriptional and translational levels, underscores the need for an in-depth analysis of it being one of the putative links between chronic inflammation and cancer.

## Abbreviations

OPLs : Oral pre-malignant lesions

OSCC : Oral Squamous Cell Carcinomas

SDS-PAGE: Sodium Dodecyl Sulphate-Polyacrylamide Gel Electrophoresis

## Competing interests

The author(s) declare that they have no competing interests.

## Authors' contributions

**AM **collected the tissue specimens and carried out all the experimental work in the study, contributed to study designing, data collection and analysis and writing of the manuscript.**SB **enrolled the patients in the study, provided the clinical specimens, patient data, follow up and clinical knowledge. **RD **enrolled the patients in the study, provided the clinical specimens, patient data and clinical knowledge. **SDG **did histopathological evaluation of all clinical specimens and assessment of immunohistochemical staining data. **RR **planned, supervised, provided financial and technical support for the study and writing of the manuscript. All authors have read and approved the final manuscript.

## Pre-publication history

The pre-publication history for this paper can be accessed here:


